# Computed tomography in acute intracerebral hemorrhage: neuroimaging predictors of hematoma expansion and outcome

**DOI:** 10.1186/s13244-022-01309-1

**Published:** 2022-11-22

**Authors:** Amir Hillal, Teresa Ullberg, Birgitta Ramgren, Johan Wassélius

**Affiliations:** 1 Department of Medical Imaging and Physiology, Skåne University Hospital, 221 85 Lund, Sweden; 2 Department of Clinical Sciences Lund, Lund University, Lund, Sweden; 3 Department of Neurology, Skåne University Hospital, Lund, Sweden

**Keywords:** Intracerebral hemorrhage, Computed tomography, Imaging, Volumetric measurement, Outcome prognostication

## Abstract

Intracerebral hemorrhage (ICH) accounts for 10–20% of all strokes worldwide and is associated with serious outcomes, including a 30-day mortality rate of up to 40%. Neuroimaging is pivotal in diagnosing ICH as early detection and determination of underlying cause, and risk for expansion/rebleeding is essential in providing the correct treatment. Non-contrast computed tomography (NCCT) is the most used modality for detection of ICH, identification of prognostic markers and measurements of hematoma volume, all of which are of major importance to predict outcome. The strongest predictors of 30-day mortality and functional outcome for ICH patients are baseline hematoma volume and hematoma expansion. Even so, exact hematoma measurement is rare in clinical routine practice, primarily due to a lack of tools available for fast, effective, and reliable volumetric tools. In this educational review, we discuss neuroimaging findings for ICH from NCCT images, and their prognostic value, as well as the use of semi-automatic and fully automated hematoma volumetric methods and assessment of hematoma expansion in prognostic studies.

## Key points

- Neuroimaging is pivotal in diagnosing intracerebral hemorrhage (ICH) and predicting its cause.
- Baseline hematoma volume and hematoma expansion at repeated imaging are the strongest prognosticators in ICH.
- Specific imaging markers on computed tomography (CT) can aid in predicting ICH prognosis including risk of hematoma expansion and/or rebleeding.
- ICH volume can be measured by manual, semi-automatic and automatic methods

## Introduction

Spontaneous intracerebral hemorrhage (ICH) represents non-traumatic bleeding within the brain parenchyma. ICH accounts for 10–20% of all acute strokes worldwide and is the most fatal type of stroke, with a 30-day mortality rate of up to 40%, and a 1 year mortality rate reaching 60% [1]. Between 1990 and 2016, the absolute number of stroke events and mortality rate increased worldwide. However, the age-standardized incidence and death rate for ICH have decreased in all regions worldwide [2]. There were 4.1 million new cases of ICH worldwide in 2016, accounting for 51% of all stroke-related deaths and 64.5 million years of healthy life lost each year [2]. A study performed on 657 patients who survived up to 3 months after an ICH to assess the neurological impairment and functional disability based on the HRQoL (Health-Related Quality of Life) concept, showed that the majority of survivors (87%) after ICH had poor HRQoL (< 1). Advanced age, higher National Institutes of Health Stroke (NIHSS) score, higher systolic blood pressure, higher baseline ICH volume, deep hematoma location, and increase in neurologic deficit during the first 72 h after ICH onset were shown to be independent predictors of poor HRQoL [3].

Rapid diagnosis of intracerebral hemorrhage and identification of its etiology is key for early treatment decisions such as blood pressure management [4], reversal of anticoagulation [5] or neurosurgery [6]. The most common neuroimaging modalities available in acute settings are non-contrast computed tomography (NCCT) and CT angiography (CTA) [7, 8]. In addition to diagnosing ICH, NCCT gives detailed information about the hematoma location, shape and the presence of intraventricular extension. NCCT and can further be used to measure the hematoma volume which is considered the strongest predictor of 30-day mortality and functional outcome for ICH patients [9]. Accurate and efficient tools for measuring hematoma volume are therefore a major medical need.

Hematoma expansion is an important risk factor for poor clinical and functional outcome and is considered modifiable if identified early. It has been shown that 30% of ICH patients develop hematoma expansion [10], which occurs mostly during the first 6 h following stroke onset [11]. There are several imaging markers shown to be able to predict early hematoma expansion and active bleeding with good accuracy on NCCT (blend sign, black hole sign, swirl sign and island sign) and CTA (spot sign) [12].

In this educational review aimed for general radiologists and neuroradiologists at different stages of training, following a short introduction to clinical characteristics and risk factors of ICH, we focus on neuroimaging findings and signs on NCCT useful for determining ICH etiology and predicting hematoma expansion and clinical outcome. We also describe the most widely used manual, semi-automatic and fully automated methods for measuring hematoma volume.

## Risk factors and clinical characteristics of ICH

Intracerebral hemorrhage can be spontaneous (also known as non-traumatic ICH), related to trauma (traumatic ICH) [13, 14]. Spontaneous ICH arises from chronic, progressive small vessel disease occurring primarily due to chronic hypertension and cerebral amyloid angiopathy [15, 16]. Other less common etiologies of non-traumatic ICH include arteriovenous or other vascular malformations, cerebral venous thrombosis, hemorrhagic transformation of ischemic stroke, mycotic aneurysms, Moya Moya disease, cerebral vasculitis, and primary or metastatic tumor. [17] (Table 1 and Fig. 3).

**Table 1:** The most common etiologies of spontaneous (non-traumatic) ICH

*Common etiologies of spontaneous ICH*
Hypertensive angiopathy
Cerebral amyloid angiopathy
*Less common etiologies*
Hemorrhagic conversion of ischemic stroke
Cerebral aneurysms
Mycotic aneurysms
Cerebral arteriovenous malformation
Cavernoma
Dural arteriovenous fistulae
Vasculitis or vasculopathy
Cortical vein- or sinus thrombosis
Tumor

*ICH* Intracerebral hemorrhage

Hypertension, cerebral amyloid angiopathy (CAA), oral anticoagulant treatment and older age are considered the most important risk factors of ICH [18]. Additional risk factors include cigarette smoking, excessive alcohol consumption, low-density lipoprotein cholesterol levels, genetics (Apolipoprotein E gene), and ethnicity (Asian ethnicity) [1, 18, 19].

Symptoms of ICH are identical to those of ischemic stroke, as patients present with sudden neurologic deficits. Presenting with decreased level of consciousness, vomiting and headache may be indicative of ICH, but only neuroimaging can determine if the stroke is ischemic or hemorrhagic [20].

In ICH, a lower Glasgow coma scale (GCS) score and higher NIHSS score are predictors of larger baseline hematoma volume, mortality, severe disability, and hematoma expansion [10, 21]. However, the CT findings may not always correlate with the clinical symptoms of patients with ICH especially in cases of lobar bleedings where large hematomas may be seen in patients with relatively mild symptoms [22]. Wagner et al. [23] found that both neurosurgeons and neurologists had limitations in clinical estimation of ICH patients, other than of GCS and NIHSS scores, concluding that a collaborative approach by neurologists and neurosurgeons in addition to careful evaluation of the imaging findings is important to make appropriate acute treatment decisions.

High systolic blood pressure (above 160 mmHg) is associated with larger initial hematoma volume, an increased risk of hematoma expansion and poor functional outcome. The mechanism of which is likely through rupture and continuous bleeding from small cerebral blood vessels [24–26].

The use of antithrombotic drugs increases the risk of hematoma expansion [27]. Oral anticoagulation treatment increases initial hematoma volume and increases the risk of hematoma expansion by 6.2 times [28]. Patients taking non-vitamin K oral anticoagulant medication prior to their ICH had smaller baseline hematomas, lower risk of expansion, and better functional outcome compared to ICH patients on vitamin K oral anticoagulant medications [29–32].

Hyperglycemia at admission is present in more than half of ICH patients [33] and associated with poor functional outcome, increased mortality regardless of prior diabetes history [34, 35] and a 2.5-fold increased risk of hematoma expansion [36].

Interestingly, hematoma expansion is more common in ICH with onset at daytime than at night, however the underlying mechanisms are not well known [37].

The use of bundles of care for ICH including rapid anticoagulant reversal, intensive blood pressure lowering, access to intensive care and neurosurgery has shown to reduce 90-day case-fatality [38].

## Predictive scores in ICH

The ICH score [39] (Table 2) is used as a prognostic tool and calculated from the patient’s presenting GCS score, age, the presence or absence of infratentorial hemorrhage, ICH volume and the presence of intraventricular hemorrhage. There are several available prognostic scores for ICH, most of them not fully validated and not widely used in routine clinical practice. Future scores need to be developed with standardized timing, preferably using more than one time point, incorporating standardized outcome assessments based on the modified Rankin Scale, and including patient-reported outcome measures [40].

**Table 2:** The intracerebral hemorrhage score and the mortality rates associated with a total score of 0-5p

Intracerebral hemorrhage score	
Variable	Points
*GCS score*	
3–4	2
5–12	1
13–15	0
*Age*	
≥ 80	1
< 80	0
*Infratentorial hemorrhage*	
Yes	1
No	0
*Volume (mL)*	
≥ 30	1
< 30	0
*Intraventricular hemorrhage*	
Yes	1
No	0

Total score	Mortality risk (%)
0	0
1	13
2	26
3	72
4	97
5	100

*GCS* Glasgow coma scale

## Neuroimaging of ICH

**Fig. 1:**
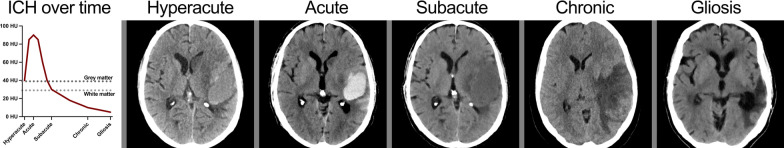
Hematoma attenuation. The left panel illustrates the changes in hematoma attenuation over time. In the *hyperacute* phase, within the first minutes of onset, the hematoma is iso-attenuating with gray matter. Within an hour, the attenuation in the hematoma increases to the hyperattenuating typical appearance of the *acute* phase, lasting for several hours. The attenuation then gradually decreases over the next days to iso-attenuating with the gray or white matter in the *subacute* phase, and then even further to become hypoattenuating in the *chronic* phase, typically within weeks of onset. Finally, the hematoma is absorbed and the lesion is healed with substance loss and *gliosis* in the surrounding brain parenchyma

NCCT is the gold standard imaging modality for the diagnosis of ICH [7] due to its availability, few contraindications and excellent sensitivity for bleeding. Although the typical finding in acute ICH is a hyperintense hematoma, the hyperacute ICH may be iso-attenuating to gray matter, rapidly increasing within minutes to the hyperintense state seen for hours to days after which the attenuation gradually decreases to be iso-attenuating to white matter in the subacute stage (days–weeks) and hypoattenuating in the chronic state over (weeks–months), as illustrated in Fig. 1.

**Fig. 2:**
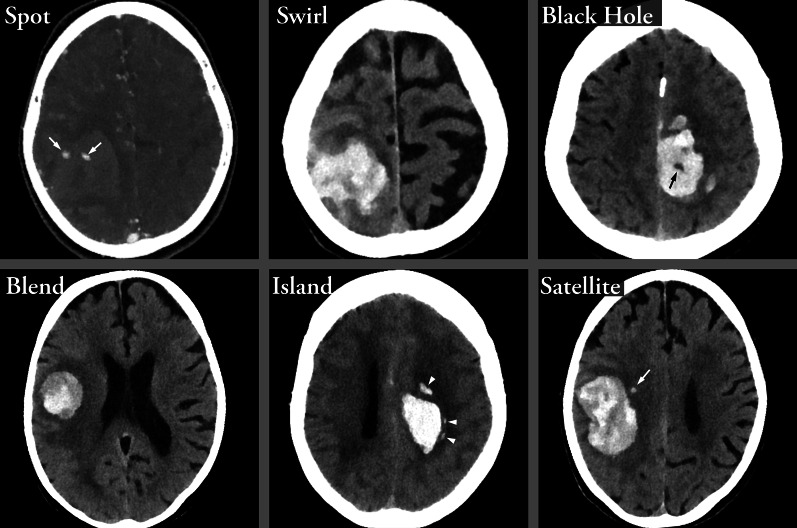
Imaging Signs. The *Spot sign* is seen on CTA, the other signs on NCCT. *Spot sign*—Spot sign is defined as one or more focal enhancements (white arrows) within an acute parenchymal hematoma on CTA, suggestive of active contrast extravasation/active bleeding into the hematoma*. Swirl sign*—The swirl sign is defined as regions of hypo-attenuation or iso-attenuation within the hyper-attenuated ICH, suggestive of hyperacute bleeding/active bleeding within the acute hematoma. *Black hole sign*—The black hole sign is defined as well-defined round or oval areas (black arrow) of hypoattenuation enclosed within the hyperattenuating hematoma, without any connection with the adjacent brain parenchyma. *Blend sign*—The blend sign is defined as regions of mixed hyper-attenuated and hypo-attenuated areas with identifiable boundaries located within the hemorrhage. *Island sign*—The island sign is defined as: (1) three or more scattered small oval or round hematomas separated from the main hematoma (white arrowheads), or (2) four or more small bubble-like or sprout-like hematomas, some or all of which may be connected with the main hematoma. *Satellite sign*—The satellite sign is defined as any small hemorrhage (white arrow) with an isolating distance of 1–20 mm from the main hematoma

NCCT can be used to diagnose intraventricular hematoma extension, hydrocephalus, presence and degree of edema, midline shift and brainstem compression secondary to the mass effect from the hematoma. Several NCCT imaging markers (blend sign, black hole sign, swirl sign and island sign, illustrated in Fig. 2) that have been shown to predict early hematoma expansion and/or active bleeding are specifically discussed below [12].

CTA is an effective modality for diagnosing the underlying causes of ICH in the acute setting [8], specifically when the cause is suspected to be secondary to vascular abnormalities such as arteriovenous malformation or other intracranial pathologies [8, 20]. The spot sign on CTA images is caused by contrast extravasation due to active bleeding within the hematoma and is therefore a predictor of hematoma growth and poor clinical outcome [41]. However, due to the additional radiation exposure, the need for of iodine contrast medium and the risk of contrast-induced nephropathy, CTA is often not routinely used in the workup of ICH [8] but the 2022 guidelines for the management of patients with spontaneous ICH from the American Heart Association/American Stroke Association suggest it is reasonable to perform CTA within the first hours of onset to identify patients at risk for subsequent hematoma expansion [42].

Magnetic resonance imaging (MRI) has similar accuracy for diagnosing acute ICH as NCCT and is superior to CT in distinguishing between ICH and hemorrhagic transformation of an acute ischemic lesion [43]. MRI gradient echo sequences can identify microbleeds within the brain parenchyma as well as blood products that represent chronic lesions and cavernomas—findings that typically cannot be diagnosed by NCCT [43]. Contrast-enhanced MRI can be performed after the hematoma resolves to identify an underlying tumor. Magnetic resonance angiography can be useful in imaging brain vessels and can be performed without contrast medium. Nonetheless, MRI is rarely used in the acute setting for ICH diagnosis due to its cost and lower availability [20].

### The role of neuroimaging for determining the ICH etiology

**Fig. 3:**
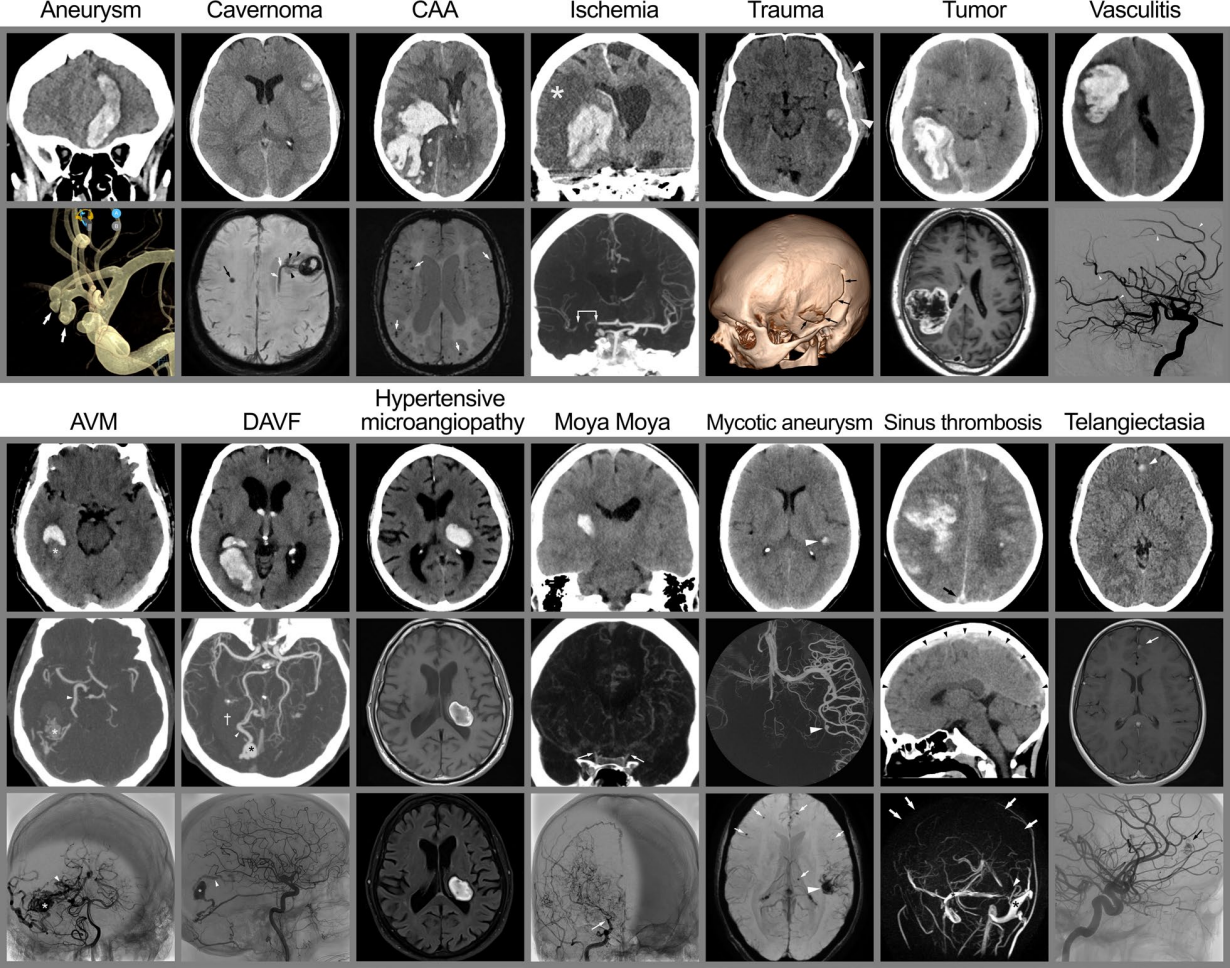
Etiology. The figure illustrates the most common etiologies of ICH. *Top row: Aneurysm*—Bleeding from cerebral aneurysms may rarely present predominantly as ICH (top panel), such as this case of a bi-lobulated anterior communicating artery aneurysm (white arrows in CBCT 3D reconstruction in the bottom panel). *Cavernoma*—Bleeding in a cavernoma in the left hemisphere (top panel) with an associated DVA (black arrowheads and white arrows for the draining vein) and another cavernoma (black arrow) in the contralateral hemisphere on MRI (SWI). *CAA (Cerebral Amyloid Angiopathy)*—with a large parietal lobar ICH with the typical “finger-like projections” (top panel) and multiple amyloid deposits throughout the brain on a preceding MRI (SWAN, bottom panel). *Ischemia*—Illustrating an ICH an association with an acute ischemic stroke (top panel) caused by an embolic occlusion of the terminal internal carotid artery (T-occlusion—indicated by the white arrows in the CTA in the lower panel). *Trauma*—A common cause of ICH, illustrated by a temporal ICH in the left hemisphere, associated with an extracranial hematoma (white arrowheads in the top panel) and a skull fracture (black arrows in the 3D reconstruction in the bottom panel). *Tumor*—A large ICH in the right hemisphere with an underlying glioma, visualized on a subsequent MRI (T1-TSE with Gadolinium in the bottom panel). *Vasculitis*—A large frontal ICH associated with CNS vasculitis with several stenosed intracranial arteries (examples indicated by white arrowheads in the sagittal DSA in the bottom panel). *Bottom row: AVM (ArterioVenous Malformation)*—A temporal ICH where the AVM nidus (asterisk in all 3 panels) is protruding into the hematoma. The main feeder is indicated by white arrowheads (middle panel CTA and bottom panel DSA). *DAVF (Dural ArterioVenous Fistula)* – ICH with intraventricular extension adjacent to a DAVF (hematoma indicated by † in the CTA; middle panel) with an ectasia (asterisks in the middle and bottom panels) on a large draining vein (white arrowheads in the middle and bottom panels). *Hypertensive microangiopathy*—Typical location in the basal ganglia as this example with an ICH in the left thalamus, shown on CT (top panel) and MRI (T1 TSE in the middle panel and T2 FLARI in the bottom panel). *Moya Moya*—ICH caused by increased demand from thalamostriatal collateral vessels due to stenosis/occlusion of the distal internal carotid arteries (white arrows in the CTA in the middle panel and DSA in the lower panel). It is the collateral network of numerous small vessels that give rise to the typical “puff of smoke” appearance in advanced stages. *Mycotic aneurysm*—illustrated by a small ICH indicated by white arrowheads on CT (top panel) and MRI (SWI in the bottom panel) from a mycotic aneurysm on a distal MCA branch (white arrowhead in the CBCT angiography in middle panel) caused by septal emboli from staphylococcal endocarditis. Additional septic emboli seen on MRI are indicated by white arrows (SWI in the bottom panel). *Sinus thrombosis*—Illustrated by bilateral ICH in a case with extensive thrombosis of the superior sagittal sinus. The hyperdense appearance on NCCT (black arrowheads) is illustrated in the middle panel and the absence of superior sagittal sinus (white arrows on MR venography, bottom panel) and the torcula (white arrowhead in the bottom panel). *Telangiectasia*—Illustrated by an Ossler patient with a small frontal ICH where MR shows gadolinium enhancement in the lesion (white arrow in the middle panel; MR T1 TSE with Gadolinium) corresponding to a small telangiectasia without shunting on DSA (black arrow in bottom panel)

There are several different underlying etiologies causing ICH, the most common are illustrated in Fig. 3.

Intracerebral hemorrhage location can predict its etiology. Deep/non-lobar (basal ganglia, thalamus, brainstem, deep cerebellum) ICHs usually occur due to long-standing hypertension in addition to other risk factors such as diabetes mellitus, male sex, alcohol overuse, and underweight [44]. Pooled results from several studies including a total of about 4000 patients showed that hypertension was twice as common in patients with deep ICH as in those with lobar ICH [45].

Chronic hypertension induces degenerative changes of small perforating arteries leading to rupture, and deep hemorrhages that can extend into the ventricles [46]. Hypertensive microangiopathy (also known as chronic hypertensive encephalopathy) accounts for up to 35% of all spontaneous ICHs [47], and it is caused by the effects of long-standing hypertension on the brain leading to lipohyalinosis and Charcot-Brouchard aneurysms that can rupture and cause hypertensive hemorrhages. Hypertensive microangiopathy causes brain microbleeds, best visualized on T2-weighted MRI sequences (specifically susceptibility-weighted imaging as small foci of susceptibility blooming in the basal ganglia, pons and cerebellar hemispheres. Presence of brain microbleeds is significantly correlated with history of hemorrhagic stroke and has been shown to be a predictor of future ICH [48, 49]. A study done on 2164 patients who had undergone 2416 consecutive brain MRI studies showed that presence of microhemorrhages had the highest significant correlation (*p* < 0.001) with history of hemorrhagic stroke. Out of 139 patients with microhemorrhages followed clinically for more than 1 month, four patients suffered new hemorrhagic strokes [48].

Cerebral amyloid angiopathy (CAA) is caused by progressive deposition of beta-amyloid on the wall of leptomeningeal and cortical vessels that results in them becoming fragile [50]. CAA is typically associated with lobar ICH and accounts for up to 20% of all spontaneous ICHs [51]. A typical imaging feature seen when using NCCT is “finger-like projections” which is included in the Edinburgh criteria (Table 3) [52] proposed by Rodrigues et al. in 2018 to identify lobar ICH associated with CAA. The presence of subarachnoid hemorrhage, in combination with either the APOE *ε*4 allele, or “finger-like projections” has a nearly 100% sensitivity in determining CAA. Conversely, the absence thereof can be used to rule out CAA for lobar ICH [52]. However, the Edinburgh criteria can only indicate a high probability of CAA and a major limitation of clinical use of the criteria is that the APOE-status is typically not known by the radiologist at image examination. Further studies on the use of the Edinburgh criteria based solely on the CT imaging features may be warranted.

**Table 3:** The Edinburgh Criteria for predicting the probability of Chronic Amyloid Angiopathy (CAA) as the underlying cause of ICH

Edinburgh criteria	
High probability of CAA	Lobar ICH with subarachnoid hemorrhage on CT
	AND
	Finger-like projections from the ICH on CT
	OR
	Possession of at least one APOE *ε*4 allele
Intermediate probability of CAA	Lobar ICH with either
	Subarachnoid hemorrhage on CT
	OR
	Possession of at least one APOE *ε*4 allele
Low probability of CAA	Lobar ICH with neither subarachnoid hemorrhage nor possession of any APOE *ε*4 allele

*CAA* Cerebral Amyloid Angiopathy, *ICH* Intracerebral Hemorrhage, *APOE* Apolipoprotein E

MRI is superior to NCCT in identifying small vessel disease, which helps distinguishing CAA from hypertensive arteriopathy. Multiple lobar cerebral microbleeds and/or cortical superficial siderosis are strong imaging predictors of CAA as described in the Boston criteria (Table 4) [51, 53, 54]. The Boston criteria was first proposed by Greenberg SM et al. in 1995 for diagnosing CAA, and is composed of clinical, imaging and neuropathological parameters [55]. The Boston criteria were later modified in 2010 by Linn et al. by incorporating cortical superficial siderosis into the radiological diagnosis of probable CAA. The modified Boston criteria have an increased sensitivity with a slight decrease in specificity compared to the original Boston criteria [54].

**Table 4:** The modified Boston criteria for predicting the probability of Chronic Amyloid Angiopathy (CAA) as the underlying cause of ICH

Modified Boston criteria	
Definite CAA	Full post-mortem examination demonstrating:
	Lobar, cortical, or cortico-subcortical hemorrhage
	Severe CAA with vasculopathy
	Absence of other diagnostic lesion
Probable CAA with supporting pathology	Clinical data and pathologic tissue (evacuated hematoma or cortical biopsy) demonstrating:
	Lobar, cortical or cortical-subcortical hemorrhage (including ICH and/or CMB)
	Some degree of CAA in specimen
	Absence of other diagnostic lesion
Probable CAA	Clinical data and MRI or CT demonstrating:
	Multiple hemorrhages (ICH, CMB) restricted to lobar, cortical, or cortico-subcortical regions (cerebellar hemorrhages allowed),
	OR
	Single lobar, cortical or cortical-subcortical hemorrhage and cSS (focal or disseminated)
	Age ≥ 55 years
	Absence of other causes of hemorrhages* or cSS
Possible CAA	Clinical data and MRI or CT demonstrating:
	Single, lobar, cortical, or cortico-subcortical ICH, CMB;
	OR
	Presence of cSS (focal or disseminated)
	Age ≥ 55 years
	Absence of other causes of hemorrhages* or cSS

*Other causes of hemorrhage (differential diagnosis of lobar hemorrhages): antecedent head trauma, hemorrhagic transformation of an ischemic stroke, arteriovenous malformation, hemorrhagic tumor, warfarin therapy with international normalization ratio > 3, and vasculitis. CAA: cerebral amyloid angiopathy; MRI: magnetic resonance imaging; ICH: intracerebral hemorrhage; cSS; cortical superficial siderosis; CMB: cerebral microbleeds

**Fig. 4:**
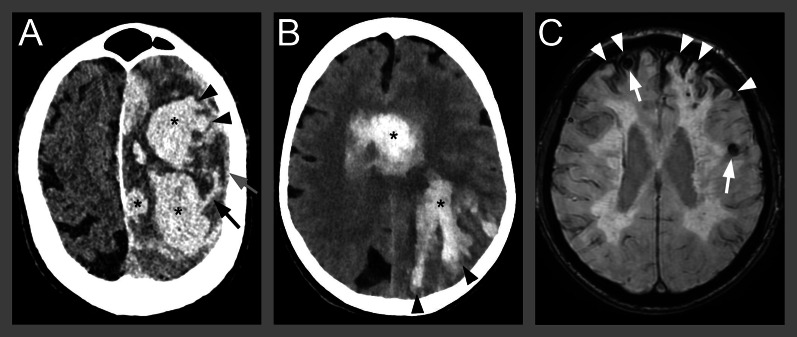
Cerebral amyloid angiopathy imaging features. Examples of imaging features of cerebral amyloid angiopathy (CAA) on CT and MRI. CT imaging predictors of CAA (panel **A** and **B**) showing ICH with multifocal components (black asterisks), finger-like projections (black arrowheads), subarachnoid hemorrhage component (black arrow) and subdural hemorrhage component (gray arrow). MRI of cerebral amyloid angiopathy (panel **C**) showing multiple lobar cerebral microbleeds (white arrows) and cortical superficial siderosis (white arrowheads)

White matter hyperintensities and enlarged perivascular spaces are predictive of CAA when located in the lobar regions, and of hypertensive arteriopathy when located in the deep and infratentorial regions [56, 57]. Susceptibility-weighted MRI can show small foci of susceptibility blooming in the cerebral white matter which aids in the clinical diagnosis of CAA [50, 58]. CT and MRI imaging features that predict CAA are shown in Fig. 4.

### The role of neuroimaging in predicting prognosis following ICH

Neuroimaging findings are of paramount importance to assess the prognosis following ICH. Examples of the most common neuroimaging predictors of ICH prognosis are shown in Table 5.

**Table 5:** Examples of the most common neuroimaging markers on NCCT (CTA for spot sign) relevant for outcome prognosticating and corresponding performance metrics and references

Neuroimaging predictors of ICH outcome			
Imaging feature	Predictive of	Performance metrics	References
Hematoma shape and density	Hematoma expansion	Shape:	[64]
		Sensitivity 69%, specificity 46%	
		Density:	
		Sensitivity 44%, specificity 72”	
Hematoma volume	Hematoma expansion, outcome and 30-day mortality	Sensitivity 96%, specificity 98%	[9]
Perihematomal edema	hematoma expansion and outcome	Odds ratio 0.59	[73]
Hematoma expansion	Outcome and mortality	Odds ratio 4.59	[10]
Intraventricular extension	hematoma expansion and outcome	Sensitivity 43%, specificity 85%	[88]
Spot sign	hematoma expansion	Sensitivity 53%, specificity 88%	[95]
Swirl sign	Hematoma expansion	Sensitivity 45%, specificity 79%	[98]
Black hole sign	Hematoma expansion	Sensitivity 30%, specificity 91%	[100]
Blend sign	Hematoma expansion	Sensitivity 28%, specificity 92%	[104]
Island sign	Hematoma expansion	Sensitivity 45%, specificity 98%	[106]
Satellite sign	Hematoma expansion	Sensitivity 54%, specificity 94%	[111]

*ICH* Intracerebral Hemorrhage

#### Hematoma volume

Hematoma volume is considered the strongest predictor of 30-day mortality and functional outcome for ICH patients [9]. The 30-day mortality rate is less than 20% for patients with hematoma volume < 30 ml, whereas volumes larger than 60 ml are associated with > 90% mortality rate. Patients with a hematoma volume of more than 30 ml are more likely to be functionally dependent at 30 days [9]. Several studies have shown that a baseline hematoma volume of 30 ml or more is a predictor of hematoma expansion and poor outcome, whereas a hematoma volume of 10 ml or less is associated with a lower probability of hematoma expansion and predictive of a favorable functional outcome [59–61].

#### Hematoma location

Lobar hemorrhages are commonly associated with CAA and feature subarachnoid extension and finger-like projections on NCCT images [52].

The INTERACT2 study showed that ICHs in the posterior limb of the internal capsule, thalamus, and infratentorial sites are associated with poor clinical outcomes. ICHs engaging specifically the thalamus and the posterior limb of internal capsule showed the highest association with death or major disability [62].

The association between hematoma volume and location was established in a study by Roh et al. [63] that showed significantly larger baseline hematoma volume for lobar ICH compared to deep ICH. In addition, hematoma expansion was found to be less common in lobar ICH compared to deep [63]. The association between larger hematoma volumes and lobar location has been confirmed in a recent study [64].

#### Hematoma shape and density

Hematoma shape was classified by Fujii et al. as either *round*, *irregular*, or *separated* [65]. Irregularly shaped hematomas have been associated with increased risk of expansion during the first 24 h of onset [66]. The association between irregular hematoma shape and early expansion has been confirmed in several studies and is also linked to larger perihematomal edema volume compared to regular-shaped hematomas [67–69].

In 2009, Barras et al. proposed a 5-point categorical scales based on hematoma shape and density. ICHs were categorized into either homogenous/regular (Category 1–2) or heterogenous/irregular (Category 3–5). It was found that median hematoma growth (10–25 ml) was significantly higher in the large, heterogenous and irregular shaped hematomas compared to the small, regular and homogenous hematomas [70].

#### Perihematomal edema

Perihematomal edema (PHE) progresses aggressively within the first 24 h and continues to increase rapidly 3 days post-ictus. The edema peaks initially at day 4–5, remains gradually elevated until day 14, then decreases [71]. Elevated oncotic pressure of the perihematomal space due to infiltration of blood components from the hematoma, along with inflammation, thrombin cascade and erythrocyte lysis products cause blood–brain barrier disruption. These factors lead to the formation of a vasogenic edema. Oxidative stress induced by vasogenic edema, and the release of cytotoxic substances can induce a secondary cytotoxic edema [72]. PHE is an independent prognostic factor for death or dependency. The attenuation of the PHE at 72 h after onset has also been shown to predict outcome, with lower attenuation values (suggestive of a more intense PHE) being predictive of a poor outcome [73]. Baseline hematoma volume is also associated with PHE growth [69]. The mean distance from the hematoma border and the outer border of the edema has been defined as the *edema extension distance* [74]. The edema extension distance at 72 h is associated with larger baseline hematoma, older age, lobar location and intraventricular hemorrhage extension [75].

#### Hematoma expansion

Hematoma growth occurs mostly within the first few hours (0–6) after onset, which makes early follow-up NCCT important for prognosis and for implementing any early interventions [11]. Early hematoma expansion has been shown to occur in 30% of ICH patients and is considered a modifiable predictor of poor outcome [10]. Sembolini et al. found that hematoma expansion was associated with a larger baseline hematoma volume, higher NIHSS score, higher cumulative mortality, and long-term functional dependence rate [76]. In the INTERACT-1 study, it was shown that hematoma growth of 1 ml was associated with a 5% increase of the risk of death or dependency [77]. The definition of hematoma expansion varies substantially between studies [65, 78, 79]. Hematoma growth has been defined in studies as either a > 33% or a ≥ 6 ml increase in volume compared to the baseline CT hematoma volume [80–82], or by an increase in volume of ≥ 12.5 ml compared to the baseline CT—the latter definition has been shown to be superior for outcome prediction [83]. The 2022 for the management of patients with spontaneous ICH from the American Heart Association/American Stroke Association suggest that repeated head CT during the first 24 h after onset can be useful to evaluate hematoma expansion [42].

#### Intraventricular hemorrhage extension and hydrocephalus

Expansion of hematomas located near the ventricular system may cause a break in the ventricular wall and a subsequent intraventricular hemorrhage (IVH). IVH is present in 45% of ICH cases, with the highest frequency for thalamic and caudate bleedings [84]. Blood within the ventricular system can cause hydrocephalus, either by obstructing the cerebrospinal fluid flow or by impairing the cerebrospinal fluid resorption at the level of the arachnoid granulations [85].

IVH is predictive of early (within 24 h) and late (up to 7 days) neurological deterioration [86] and poor outcome [87, 88]. IVH is also strongly associated with pre-hospital loss of consciousness [89]. It has been shown that the prognostic value of hematoma expansion is even higher if the IVH component is also included [90].

### Specific NCCT and CTA imaging markers

There are several specific imaging signs associated with the risk of hematoma expansion and prognosis. Examples of such imaging signs on NCCT and CTA are shown in Fig. 2.

#### Spot sign

Spot sign is defined as one or more foci of contrast enhancement within an acute parenchymal hematoma on a CTA image. A positive spot sign indicates active bleeding and is a strong predictor for hematoma expansion and predicts poor clinical outcome [11, 91, 92]. The occurrence of a spot sign is most common within 3 h of symptom onset, but its accuracy in predicting hematoma expansion remains high regardless of time from symptom onset [93]. Spot sign on the initial CTA image has been correlated with larger hematoma volume and increased risk of hematoma expansion on 24 h follow-up imaging [94]. A meta-analysis has shown that a positive spot sign can predict hematoma expansion with 53% sensitivity and 88% specificity [95].

#### Swirl sign

The swirl sign is defined as regions of hypo-attenuation or iso-attenuation within the hyper-attenuated ICH on NCCT images [96] and represents active bleeding within the hematoma. The swirl sign has been associated with larger hematoma size, higher frequency of midline shift, IVH extension, and hematoma expansion where the median absolute hematoma growth was 35.6% higher in patients with positive swirl sign [96, 97]. A meta-analysis has shown that the swirl sign predicts hematoma expansion with 45% sensitivity and 79% specificity [98].

#### Black hole sign

The black hole sign is defined as a round or oval-like shaped area of hypoattenuation enclosed within the hyperattenuating hematoma, without any connection with the adjacent brain parenchyma on NCCT images. It represents mixed density—and thereby bleeding of different ages—within the same hematoma, suggestive of bleeding at several timepoints and therefore predictive of hematoma expansion [99]. The hypo-attenuated area is described as having a density difference of at least 28 Hounsfield units compared to the hyper-attenuated hematoma [100]. A meta-analysis has shown that the black hole sign predicts hematoma expansion with a 30% sensitivity and 91% specificity [101].

#### Blend sign

The blend sign on NCCT images was defined by Li et al. [102] in 2015 as a region of mixed hyper-attenuated and hypo-attenuated areas with identifiable boundaries located within the hemorrhage. The blend sign is associated with hematoma expansion and poor outcome [103] as well as increased risk of postoperative rebleeding in patients undergoing minimally invasive neurosurgery [104]. In a meta-analysis, the blend sign has been shown to predict hematoma expansion with a sensitivity of 28% and specificity of 92% [105].

#### Island sign

The island sign was proposed by Li et al. in 2017 as a predictor of hematoma expansion. It is defined as [1] three or more scattered small oval or round hematomas all separate from the main hematoma [2]; or four or more small bubble-like or sprout-like but not lobulated hematomas, some or all of which could be connected with the main hematoma. Island signs have been proposed to be associated with multiple bleeding sources caused by injury of adjacent arterioles, possibly by shear force from the initial hematoma. The island sign has been shown to be present in as many as 44.7% of expanding hematomas and is a highly specific (98.2% specificity) with a 44.7% sensitivity for predicting hematoma expansion [106], and it’s prognostic values has been confirmed by several other studies [107–110].

#### Satellite sign

The satellite sign is defined as any small hemorrhage with an isolating distance of 1–20 mm from the main hematoma and is predictive of expansion with 54% in sensitivity and 94% specificity [111].

**Fig. 5:**
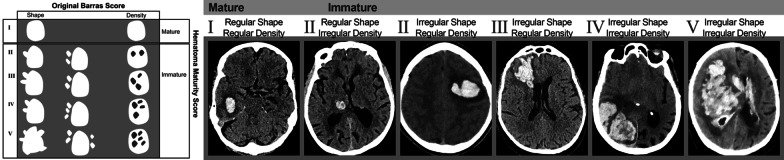
Hematoma Maturity Score. Examples of the two categories of Hematoma Maturity Score. Categories of Hematoma Maturity Score classified ICH as mature (completely homogeneous in density and shape) and immature (ICH with any irregularity in density or shape) illustrated by typical cases. Categories of the Barras score classified ICH as homogenous and regular if the Barras score was < 4, and heterogenous and irregular if the original Barras score of the variables was ≥ 4. The left panel illustrating the score is adopted from the original publication by Serrano et al. [112]

Serrano E et al. compared two combined indicators—Combined Barras Total Score and Hematoma Maturity Score—to individual radiological NCCT signs (Black hole, blend, island, swirl, Barras classification, any hypodensity, any irregularity) (Fig. 5) to predict functional outcome (according to the modified Rankin Scale) of 114 ICH patients [112]. The Hematoma Maturity Score categorized ICH into Mature: completely homogenous without any irregularity or hypodensity, and Immature: any irregularity in density and shape. Categories of the original Barras classification were grouped into a one Combined Barras Total Score, where ICH was classified as homogenous and regular if the original Barras score was < 4, and ICH was considered heterogenous and irregular if the original Barras score of the variables was ≥ 4. Results showed that among all evaluated radiological signs, The Hematoma Maturity Score (Immature) had the highest accuracy (AUC 0.779) for the prediction of poor outcome for ICH patients, followed by Combined Barras Total Score ≥ 4 (AUC of 0.727) [112].

## Volumetric assessment methods

**Fig. 6:**
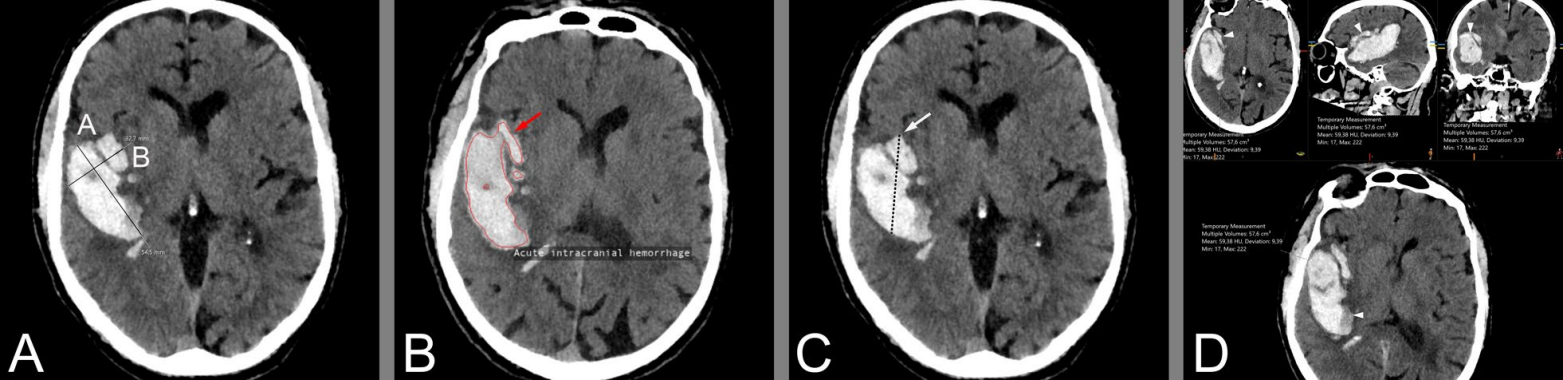
Volumetric methodology. Panel **A** illustrates the principle of the ABC/2 method where **A** is the largest diameter of the hematoma in axial images, **B** is the largest perpendicular diameter, and **C** is the number of slices with hematoma visible multiplied with the slice distance. Panel **B** illustrates the measurement done by the automated hematoma segmentation tool qER-qQuant from Qure.ai. Panel **C** and **D** illustrate the semi-automated measurement done in Sectra IDS7 where an arbitrary diameter is drawn through the hematoma (panel **C**) and the software then identifies the boundaries and calculate the volume (panel **D**)

Examples of volumetric assessment methods are shown in Fig. 6.

### Manual volume assessment

Manual segmentation can be performed in most Picture Archiving and Communication Systems by measuring the hematoma area (typically using area measurement tools) in consecutive images and multiply the total area with the slice thickness. However, such manual segmentation is time consuming and therefore rarely used in routine health care.

The TADA formula, commonly referred to as “*ABC*/2”, where *A* is the largest diameter of the hematoma as measured on axial images; *B* is the largest diameter perpendicular to A on the same image slice, and *C* is the number of slices in which the hematoma is seen, multiplied by the slice thickness. This formula was introduced by Kwak et al. [113] and Broderick et al. [114] and was validated by Kothari et al. [115], who proposed a modified *ABC*/2 (m*ABC*/2). In the modified method each image slice containing the hematoma was compared to the image slice containing the largest hematoma area in order to estimate “*C*”. Here, each image slice where the hematoma area was > 75% of the largest area was weighted as 1. A weighting of 0.5 was given to slices where the hematoma area ranged between 25 and 75% of the largest hematoma area. Slices with a hematoma area < 25% of the largest area were excluded. The sum of these weighted slices multiplied by slice thickness determined the value of C [115].

**Fig. 7:**
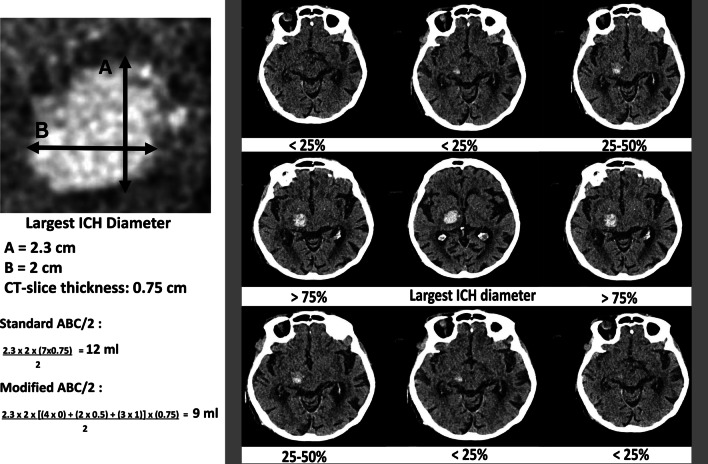
ABC/2 method variants. Example of the standard ABC/2 and modified ABC/2 manual assessment of the hematoma volume

In general, the ABC/2 methods assume an ellipsoid-shaped hematoma, leading to both under- and overestimation of the true hematoma volume in non-ellipsoid-shaped hematomas [116–119]. The modified ABC/2 method was shown to underestimate hemorrhage volumes compared to standard ABC/2 and computer-assisted semi-automatic segmentation [116], as seen in Fig. 7. In addition, the larger and the more irregular in shape the hematoma was, the larger the difference is between the techniques [116]. Maeda et al. found that the ABC/2 method *underestimated* the ICH volume by 15% compared to planimetric methods [119]. The *underestimation* was confirmed by Hussein et al. who found that for hematomas smaller than 20 ml, there was a 10% *underestimation* of ICH volume when using the ABC/2 method compared to a computer-based analysis. This *underestimation* increased to 37% for hematomas larger than 40 ml [120]. Contrary to this, Wang et al. found a systematic 10% *overestimation* of the volume for hematomas smaller than 20 ml when using the ABC/2-formula compared to a computer-assisted volumetric analysis. This *overestimation* increased to 17% for hematomas between 20 and 40 ml, and 37% for hematomas larger than 40 ml [121].

In a study on vitamin K oral anticoagulant treated patients, Huttner et al. showed that more than half of the hematomas were irregularly shaped and for these, the ABC/2 method significantly *overestimated* the hematoma volume compared with computer-assisted planimetric analysis and that modifying the denominator to ABC/3 in irregularly shaped hematomas, increased the accuracy [122]. Scherer et al. compared the ABC/2 method to manual segmentation and a novel semi-automatic threshold-based region growing tool and found substantial agreement between manual and automatic segmentations and confirmed a systematic volume overestimation by the ABC/2 formula [123]. Using a different volume measurement software (Quantomo 5, Cybertrial, Inc, Calgary, Canada), Dowlatshahi et al. [124] found the volumetric measurements to be accurate for smaller hematomas, but less so for hematomas larger than 43.8 ml. The discrepancies between studies resulting in both under- and overestimation by the *ABC*/2-method is a major concern. With access to accurate and effective methods for volumetric measurement, the *ABC*/2 method appear obsolete and will likely be less used in the future.

### Semi-automated volume measurements

Several studies have investigated computer-assisted semi-automated methods that use Hounsfield unit segmentation to estimate hematoma volume from images obtained by NCCT [125–127]. Another computer-aided measuring tool used in radiology is the Sectra Volume Measurement tool (Sectra IDS7, Sectra, Linköping, Sweden), where a line is manually drawn between two margins of the hematoma, and the software identifies the margins of the entire hematoma and calculates the volume.

### Fully automated ICH identification and volume measurement

Recently, several fully automated imaging analysis tools based on machine learning algorithms such as Rapid AI (Ischemia view Inc., Menlo Park, California, United States.), CINA® ICH (Avicenna.AI, La Ciotat, France), and qER/qQuant (Qure.ai, Mumbai, India) have been shown to accurately identify ICHs from NCCT images [128–130]. The triage and notification tool qER has been shown to accurately identify intracranial bleeds, cranial fractures, mass effect or midline shift from NCCT images (Fig. 2) [129]. The qQuant tool can be used to automatically quantify ICH volume.

The Rapid ICH module has been shown to detect various types of ICH (excluding hemorrhagic transformations) with a sensitivity of 95% and specificity of 94% within less than 2 min from NCCT images. In a study by Heit et al. [128] in 2021, ICH volumes measured by Rapid ICH correlated well with expert manual segmentation (correlation coefficient *r* = 0.983). However, the Rapid ICH volumetric quantification tool is currently only available for investigational use.

Another recently developed fully automated image analysis tool based on machine learning algorithms is the CINA® ICH software included in Canon’s ^AUTO^Stroke package. The CINA® software does not measure hematoma volume, but can detect ICH with 95.6% accuracy, 91.4% sensitivity, and 97.5% specificity [130]. A study by Rava et al. showed that the CINA® software can detect intracranial hemorrhage (intraparenchymal, intraventricular, subdural or subarachnoid) with an overall sensitivity of 93 ± 3%, and an overall specificity of 93 ± 1% [131]. However, these studies identified limitations in the software’s ability to detect small hemorrhages (< 5 mm).

## Conclusion

In this educational review aimed primarily for radiologists and neuroradiologists, we provide an overview of the most important neuroimaging findings for determining etiology and predicting outcome for ICH and describe the most widely used manual, semi-automated and automated methods for volumetric measurement for ICH from NCCT and CTA images. Identifying imaging markers on NCCT (blend sign, black hole sign, swirl sign and island sign) and CTA (spot sign) are important for prediction of early hematoma expansion and should be considered in the radiology report. Hematoma volume is the strongest predictor of 30-day mortality and functional outcome for patients with ICH and should as such be included in in the radiology report; hence, rapid and accurate tools for measuring ICH volume will become a major advancement in routine healthcare. The commonly used ABC/2 method has been shown to both under- and overestimate the volume of large and irregularly shaped ICHs as is therefore a poor substitute to accurate volume segmentation.

Future studies to assess and validate the ability of novel imaging tools to accurately identify and measure hematoma volume, perihematomal edema volume and hematoma expansion are warranted.

## Abbreviations

APOE: Apolipoprotein E
CAA: Cerebral amyloid angiopathy
CT: Computed tomography
CTA: Computed tomography angiography
DALYs: Disability-adjusted life years
DICOM: Digital Imaging and Communications in Medicine
GCS: Glasgow coma scale
ICH: Intracerebral hemorrhage
IVH: Intraventricular hemorrhage
MRI: Magnetic resonance imaging
NCCT: Non-contrast computed tomography
NIHSS: National Institutes of Health Stroke Scale
PHE: Perihematomal edema
